# Vitamin D and calcium are required at the time of denosumab administration during osteoporosis treatment

**DOI:** 10.1038/boneres.2017.21

**Published:** 2017-10-10

**Authors:** Yukio Nakamura, Takako Suzuki, Mikio Kamimura, Kohei Murakami, Shota Ikegami, Shigeharu Uchiyama, Hiroyuki Kato

**Affiliations:** 1 Department of Orthopedic Surgery, Shinshu University School of Medicine, Matsumoto, Japan; 2 Department of Orthopedic Surgery, Showa-Inan General Hospital, Komagane, Japan; 3 Center of Osteoporosis and Spinal Disorders, Kamimura Orthopaedic Clinic, Matsumoto, Japan

## Abstract

To evaluate the differences in outcomes of treatment with denosumab alone or denosumab combined with vitamin D and calcium supplementation in patients with primary osteoporosis. Patients were split into a denosumab monotherapy group (18 cases) or a denosumab plus vitamin D supplementation group (combination group; 23 cases). We measured serum bone alkaline phosphatase (BAP), tartrate-resistant acid phosphatase (TRACP)-5b and urinary N-terminal telopeptide of type-I collagen (NTX) at baseline, 1 week, as well as at 1 month and 2, 4, 8 and 12 months. We also measured bone mineral density (BMD) of L1–4 lumbar vertebrae (L)-BMD and bilateral hips (H)-BMD at baseline and at 4, 8 and 12 months. There was no significant difference in patient background. TRACP-5b and urinary NTX were significantly suppressed in both groups from 1 week to 12 months (except at 12 months for NTX). In the combination group, TRACP-5b was significantly decreased compared with the denosumab monotherapy group at 2 and 4 months (*P*<0.05). BAP was significantly suppressed in both groups at 2–12 months. L-BMD significantly increased at 8 and 12 months (8.9%) in the combination group and at 4, 8 and 12 months (6.0%) in the denosumab monotherapy group, compared with those before treatment. H-BMD was significantly increased in the combination group (3.6%) compared with the denosumab group (1.2%) at 12 months (*P*<0.05). Compared with denosumab monotherapy, combination therapy of denosumab with vitamin D and calcium stopped the decrease in calcium caused by denosumab, inhibited bone metabolism to a greater extent, and increased BMD (especially at the hips).

## Introduction

Osteoporosis is a widespread skeletal disorder that necessitates long-term care and management. The purpose of osteoporosis treatment is the prevention of fractures to maintain the activities of daily living and to thereby reduce the risk of morbidity and mortality.

Therapies for osteoporosis are based on an understanding of bone biology. Receptor activator of nuclear factor-kappa B ligand (RANKL) is a cytokine that is essential for the differentiation, activation, and survival of osteoclasts. Denosumab is a fully human monoclonal antibody against RANKL that has been shown to selectively inhibit osteoclastogenesis. Consequently, denosumab abrogates bone resorption, increases bone mineral density (BMD), and prevents fragility fractures.^1,2^ The 1-year open-label extension of the Denosumab Fracture Intervention Randomized Placebo Controlled Trial (DIRECT) demonstrated that the prevalence of non-vertebral fractures decreased for ≤10 years after denosumab treatment and that BMD increased linearly.^3^ In 2016, our research team reported that denosumab can increase BMD even in bisphosphonate (BP)-unresponsive cases.^4^ Thus, denosumab is considered to be one of the best therapeutic options for osteoporosis with respect to increased BMD, an improvement in markers of bone turnover, and the prevention of bone fractures.

In 1999, the Japanese Ministry of Health published Guidelines for the Prevention and Treatment of Osteoporosis. Those guidelines stated that whenever a placebo group is used as a control group against a drug group, sufficient calcium and vitamin D should be administered as baseline treatment. Thus, calcium addition and vitamin D supplementation is used in studies carried out in Japan and overseas.

The term “vitamin D” is an imprecise term that refers to one or more members of a group of steroid molecules. With respect to the metabolism of vitamin D, “active” vitamin D, such as calcitriol [1,25-dihidroxycholecalciferol:1, 25(OH)_2_D_3_], regulates calcium metabolism.^5^ “Native” vitamin D (which is a nutritious vitamin) is cholecalciferol. The latter is hydroxylated in the liver to become 25(OH)D_3_ (calcifediol). Then, 25(OH)D_3_ is hydroxylated in the kidney to become active vitamin D.^6^ Another type of active vitamin D, 1*α*-hydroxycholecalciferol [1*α*(OH)D_3_], has been approved in Japan as an anti-osteoporosis drug: alfacalcidol (ALF).^7^

The type of vitamin D used in research has been generally native vitamin D. In a phase-III study of denosumab in Japan, calcium (600 mg) and native vitamin D (400 IU) were used.^8^ In Japan, after approval of denosumab use, calcium and vitamin D have been recommended to prevent hypocalcemia in osteoporosis treatment using denosumab. Thus, Denotas Chewable® (calcium and vitamin D supplementation) has been recently approved for use with denosumab treatment.

Hypocalcemia is considered to be one of the most common adverse effects in denosumab treatment for osteoporosis^9,10^ (probably because of its strong anti-resorptive function in bone). However, the mechanism of bone turnover by which hypocalcemia occurs (which may or may not include vitamin D) is not known. Okada *et al.* reported that denosumab can cause hypocalcemia.^10^ Others have reported that calcium and vitamin D are recommended to take together during denosumab administration in osteoporosis treatment.^3,11^ We investigated whether supplementation with vitamin D has additive effects on markers of bone metabolism and BMD in Japanese patients with osteoporosis.

## Materials and methods

First, 45 patients were recruited from Shinshu University School of Medicine and Showa-Inan General Hospital between June 2014 and August 2015. The inclusion criteria for the study were primary osteoporotic treatment-naive patients with low bilateral hip BMD (less than −3.0 s.d.).The exclusion criteria in this study were chronic renal failure (estimated glomerular filtration rate (eGFR) <40 (mL·min^−1^ per 1.73 m^2^)), bone metabolic disorders or diabetes mellitus, which affect osteoporosis. Patients were divided into two groups: 21 cases in the denosumab monotherapy group (treated with denosumab alone) and 24 cases in the combination group (treated with denosumab and vitamin D supplementation). The patient selection was performed by simple randomization using an enveloped method. Of 45 cases, 3 cases in the denosumab monotherapy group and 1 case in the combination group were excluded from this study because they did not re-visit our institutions after the initial administration of denosumab. Finally, 41 enrolled patients completed the study (18 in the denosumab monotherapy group and 23 in the combination group; Table 1). All patients were diagnosed as having primary osteoporosis. Eight of 18 and 10 of 23 patients were pretreated with a BP before denosumab treatment (Table 1). The diagnosis of primary osteoporosis was made in accordance with revised criteria established by the Japanese Society of Bone and Mineral Research.^12^ Each patient received denosumab (60 mg, subcutaneous injection) once every 6 months in both groups. In the combination group, we gave vitamin D supplementation tablets, which are newly approved as a drug (762.5 mg of precipitated calcium carbonate, 200 IU of cholecalciferol, and 59.2 mg of magnesium carbonate), twice daily to all patients after denosumab administration.

**Figure 1:**
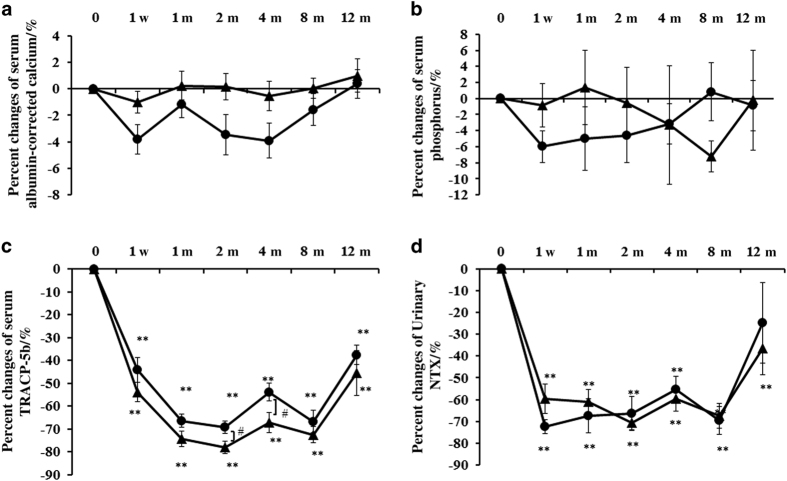
Percent changes in serum albumin-corrected calcium (Ca), phosphorus (P), and percent changes in serum TRACP-5b and urinary NTX. Percent changes in Ca (**a**) or P (**b**) did not show a significant difference between the groups. There was no significant change in Ca or P at each time point compared with those before treatment. Percent changes in serum Ca substantially decreased in the denosumab monotherapy group but did not decrease in the combination group. Percent changes in TRACP-5b levels significantly decreased at each time point in both groups compared with those before treatment. There were significant differences at 2 and 4 months between the groups (*P*<0.05). The decrease in percent change of TRACP-5b levels tended to return to the baseline level at 4 and 12 months (**c**). Percent changes in urinary NTX significantly decreased at each time point except at 12 months in the denosumab monotherapy group compared with those before treatment. There was no significant difference in urinary NTX between the groups. The decreased percent change of urinary NTX tended to return to the baseline level at 4 and 12 months (**d**). The closed circles show the denosumab monotherapy group, whereas the closed triangles show the combination group. Double asterisks denote significant differences (*P*<0.01) at 1 week, and 1, 2, 4, 8 and 12 months compared with pre-treatment in either the denosumab monotherapy or combination groups. The hashtag shows significant differences (*P*<0.05) between the denosumab monotherapy and combination groups at each time point.

**Figure 2:**
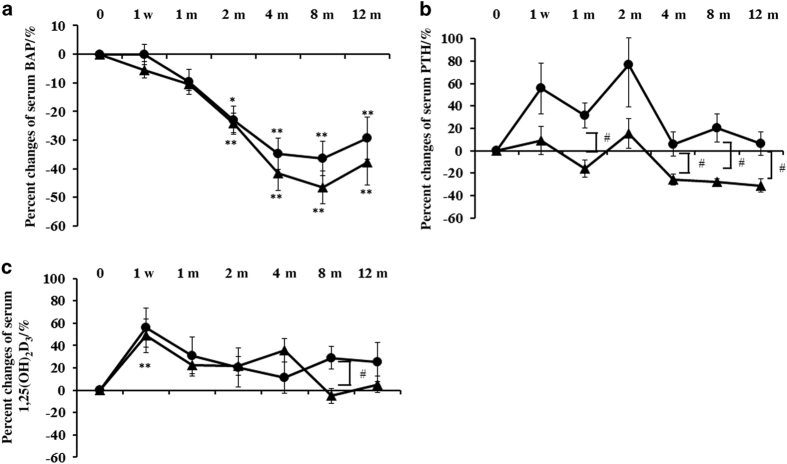
Percent changes in serum BAP, parathyroid hormone 1–84 (PTH), and 1,25(OH)_2_D_3_. Percent changes in serum BAP decreased significantly at 2–12 months in both groups (**a**). Percent changes in serum PTH increased in the denosumab monotherapy group but were mostly maintained around the baseline level in the combination group. There were significant differences at at 1, 4, 8 and 12 months between the two groups (**b**). Percent changes in serum 1,25(OH)_2_D_3_ in the denosumab monotherapy group significantly increased at 1 week compared with those before treatment and then returned to the baseline level. There was a significant difference at 8 months between the two groups (**c**). The closed circles represent the denosumab monotherapy group, whereas the closed triangles represent the combination group. Double asterisks or an asterisk denote significant differences (*P*<0.01 or *P*<0.05, respectively) at at 1 week, and 1, 2, 4, 8 and 12 months, compared with pre-treatment in either the denosumab monotherapy group or the combination group. The hashtag shows significant differences (*P*<0.05) between the denosumab monotherapy and the combination groups at each time point.

Percent changes in serum calcium, phosphorus, and markers of bone turnover are shown in Figures 1 and 2. Percent changes in serum bone alkaline phosphatase (BAP) were measured as bone-formation markers using a chemiluminescent enzyme immunoassay and antibody radioimmunoassay. Percent changes in serum tartrate-resistant acid phosphatase (TRACP)-5b and urinary N-terminal telopeptide of type-I collagen (NTX) (Osteomark; Osteox International, Seattle, WA, USA) were measured as markers of bone resorption using an enzyme-linked immunosorbent assay. Percent changes in serum parathyroid hormone 1–84 (PTH) and the active form of vitamin D [1,25(OH)_2_D_3_] were measured with immunoradiometric assays. Each marker was measured just before denosumab administration, at 1 week, as well as at 1, 2, 4, 8 and 12 months of denosumab treatment. After an overnight fast, serum and first-void urine samples were collected between 8:30 a.m. and 11:00 a.m. Immunoassays were carried out by SRL (Tokyo, Japan).

Percent changes of BMD were measured using a dual-energy X-ray absorption fan-beam bone densitometer (Lunar Prodigy; GE Healthcare, Waukesha, WI, USA) at L1–4 levels of the posteroanterior spine and bilateral hips. The coefficients of variation (CV) of the BMD measurements at the lumbar spine and hip were 0.7% and 1.1%, respectively.^13^ The least significant changes of these measurements were 1.6% and 1.5%, respectively.^14^

The study protocol was approved by the Ethics Committee of Shinshu University School of Medicine (Matsumoto, Japan) and Showa-Inan General Hospital (Komagane, Japan). This study was carried out in accordance with the ethical standards in the Declaration of Helsinki (2014 revision). The clinical trial registration number is NCT02156960, and the date of registration was 31 May 2014. Written informed consent was obtained from all patients.

## Results

There was no significant difference in patient background in either group (Table 1). Serious adverse events such as hypocalcemia or bone fracture did not occur during the study.

### Serum albumin-corrected levels of calcium and phosphorus

Percent changes in serum calcium after treatment did not change significantly between the two groups or at any time compared with those before treatment (Figure 1a). Percent changes in serum calcium substantially decreased in the denosumab monotherapy group but did not decrease in the combination group.

Percent changes in the serum phosphorus after treatment did not change significantly between groups or at any time compared with those before treatment (Figure 1b). However, the extent of percent changes in phosphorus serum levels was much less in the combination group than those in the denosumab monotherapy group in the early phase.

### Markers of bone turnover

#### Markers of bone resorption

Percent changes in serum TRACP-5b were significantly suppressed in both groups from 1 week to 12 months. In the combination group, percent changes in serum TRACP-5b were significantly decreased compared with those in the denosumab monotherapy group at 2 and 4 months (*P*<0.05; Figure 1c).

Percent changes in urinary NTX significantly decreased at each time point except at 12 months in the denosumab monotherapy group compared with those before treatment. There was no significant difference in percent changes between the two groups (Figure 1d). The decreased percent changes in serum TRACP-5b and urinary NTX tended to return to the baseline level at 4 and 12 months.

#### Markers of bone formation

Percent changes in serum BAP significantly decreased at 2–12 months and were maintained at 2–8 months in both groups (Figure 2a). After 4 months, the inhibitory effects in the combination group tended to be greater than those in the denosumab monotherapy group.

### Serum PTH and 1,25(OH)_2_D_3_

Percent changes in serum PTH increased in the denosumab monotherapy group, whereas they were maintained around the baseline level in the combination group, during the study period. Significant differences were noted at 1, 4, 8 and 12 months between the two groups (Figure 2b).

Percent changes of serum 1,25(OH)_2_D_3_ were significantly increased at 1 week only in the combination group. A significant difference was noted at 8 months between the two groups (Figure 2c).

### BMD at the lumbar spine (L-BMD) and hip (H-BMD)

**Figure 3:**
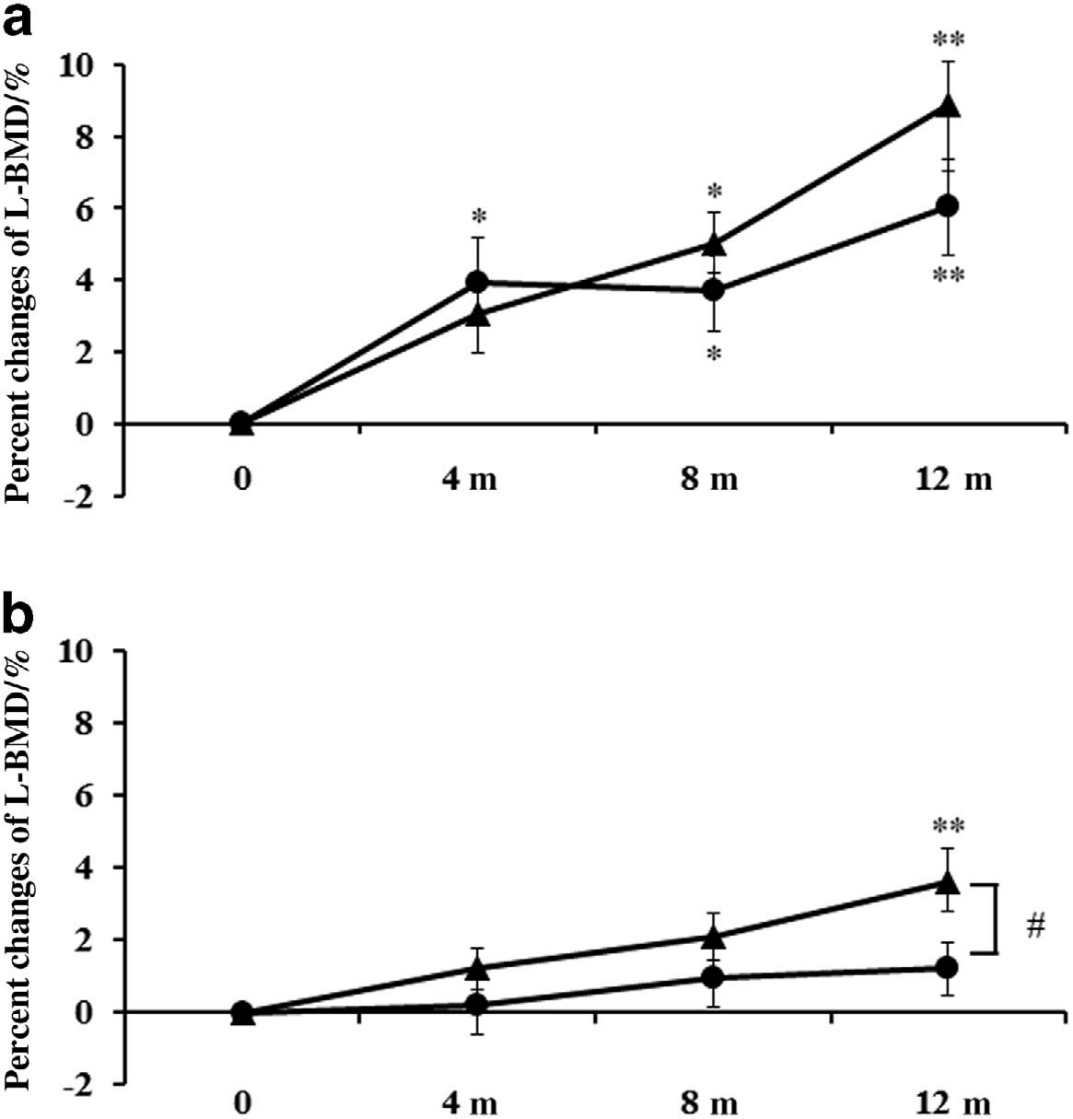
Percent changes in L-BMD and H-BMD. Percent changes of L-BMD steadily increased for 12 months in the denosumab monotherapy group (6.0% increase at 12 months) and in the combination group (8.9% increase at 12 months). There was no significant difference between the groups. In the denosumab monotherapy group, there were significant differences at 4, 8 and 12 months, whereas there were significant differences at 8 and 12 months in the combination group, compared with those before treatment (**a**). Percent changes in H-BMD steadily increased for 12 months in the denosumab monotherapy group (1.2% increase at 12 months) whereas percent changes in H-BMD increased ≤12 months in the combination group (3.6% increase at 12 months). There was a significant difference at 12 months between the two groups. In the denosumab monotherapy group, there was no significant difference, but there was a significant difference at 12 months in the combination group, compared with those before treatment (**b**). The closed circles represent the denosumab monotherapy group, whereas the closed triangles represent the combination group. Double asterisks or an asterisk denote significant differences (*P*<0.01 or *P*<0.05, respectively) at 4, 8 and 12 months, compared with pre-treatment in either the denosumab monotherapy group or the combination group. The hashtag shows significant differences (*P*<0.05) between the denosumab monotherapy and combination groups at each time point.

Percent changes in L-BMD steadily increased for 12 months in the denosumab monotherapy group (6.0% increase at 12 months) and in the combination group (8.9% increase at 12 months). There was no significant difference between the two groups (*P*=0.22). In the denosumab monotherapy group, there were significant differences of L-BMD at 4, 8 and 12 months, whereas there were significant differences at 8 and 12 months in the combination group, compared with those before treatment (Figure 3a).

Percent changes in H-BMD steadily increased for 12 months in the denosumab monotherapy group (1.2% increase at 12 months), whereas they significantly increased at 12 months in the combination group (3.6% increase at 12 months; Figure 3b). Percent changes in H-BMD significantly increased in the combination group compared with those in the denosumab monotherapy group at 12 months (*P*<0.05).

## Discussion

We report, for the first time, comparative data between denosumab treatment with or without vitamin D supplementation in Japanese patients with primary osteoporosis. Compared with denosumab monotherapy, combination therapy of denosumab with vitamin D and calcium: (i) stopped the decrease of serum calcium caused by denosumab; (ii) inhibited bone metabolism to a greater extent; (iii) inhibited the increase of serum PTH; and (iv) increased percent changes of BMD (especially at the hips).

Denosumab is a potent anti-resorptive agent. In the DIRECT carried out in Japan, Sugimoto *et al.* reported that all patients who took daily supplements containing ≥600 mg calcium and 400 IU vitamin D had a significantly decreased risk of vertebral fracture and no hypocalcemia when taking denosumab for 3 years.^3^ However, studies focusing on the effectiveness and/or adverse effects of denosumab with or without vitamin D supplementation in osteoporosis are lacking.

Body *et al.* reported that denosumab without calcium and vitamin D causes significant hypocalcemia, but the denosumab regimen was 120 mg every 4 weeks and the patients had metastatic bone disease.^11^ Our results showed that no hypocalcemia occurred in the denosumab monotherapy group, and that no serious adverse effects occurred in the denosumab monotherapy group or the combination group. The addition of vitamin D and calcium did not decrease serum calcium (which was observed in the denosumab monotherapy group). These results suggest that hypocalcemia could be prevented after denosumab treatment by supplementation with vitamin D.

Previously, we reported changes in bone turnover in the early phase after treatment with ibandronate (IBN) alone or with IBN plus ALF.^15^ Serum PTH significantly increased at 12–20 weeks in the IBN group, but ALF addition eliminated these significant changes.^15^ Shiraki *et al.* provided comparative data on ALF monotherapy and alendronate (ALN) monotherapy.^16^ Serum calcium significantly decreased in the ALN group and also increased in the ALF group, although not significantly.^16^ Serum PTH significantly decreased in the ALF group, whereas they significantly increased in the ALN group.^16^ We speculate that BP therapy decreases serum calcium by inhibition of bone metabolism (and thereby increases serum PTH) and that an analog of vitamin D [1*α*(OH)D_3_], which is converted to 1,25(OH)_2_D_3_ in the liver, may stop the increases in serum calcium and PTH.

Ebina *et al.* recently reported that denosumab plus ALF combination therapy significantly increased the femoral neck BMD values, compared with those treated with denosumab plus native vitamin D.^17^ In the present study, serum PTH were significantly increased and serum calcium were decreased only in the denosumab monotherapy group. We speculate that vitamin D supplementation did not cause the increases in serum PTH because changes in serum calcium ceased.

Patients in the combination group had increased bone-inhibitory effects compared with those in the denosumab monotherapy group. Olmos *et al.* reported that, in osteoporosis patients treated with ALN and 25(OH)D_3_ (calcifediol), cases with sufficient 25(OH)D_3_ levels showed no significant difference in the inhibition of bone metabolism. They also reported that patients with insufficient levels of 25(OH)D_3_ showed significant inhibitory effects on bone metabolism when treated with 25(OH)D_3_ in the case of ALN treatment.^18^ Our previous study showed that IBN addition inhibited bone turnover significantly more in the IBN monotherapy group than in the IBN plus ALF group.^15^ Vitamin D deficiency generally causes increased serum PTH. In addition, several studies have shown that ALF administration decreases serum PTH.^16,18^ It has been shown that PTH receptor signaling in osteoblasts and osteocytes can increase the ratio of RANKL: osteoprotegerin (the decoy receptor of RANKL) to increase the recruitment and activity of osteoclasts and hence can stimulate bone resorption.^19^ Thus, the inhibitory effects of PTH caused by the administration of vitamin D might have resulted in greater inhibition in the combination group than in the denosumab monotherapy group.

In the present study, denosumab administration increased L-BMD values ≤6.0% and H-BMD values ≤1.2% at 12 months. Vitamin D supplementation increased L-BMD values ≤8.9% and H-BMD values ≤3.6% at 12 months. A greater significant difference in H-BMD values at 12 months in the combination group compared with those in the denosumab monotherapy group was noted. Ebina *et al.* have reported that the PTH values were significantly lower in the native vitamin D group than in the ALF group, which had shown BMD increased effects, although there was no difference in the bone turnover inhibitory effects between both groups.^17^ On the basis of the findings of Ebina *et al.*^17^ and our findings in this study, it is conceivable that the vitamin D addition significantly increased BMD values potentially due to the decrease of the serum increased PTH caused by denosumab treatment.

Leslie *et al.* reported that treatment-related increases in H-BMD are associated with a reduced risk of fracture compared with BMD, whereas decreases in BMD are associated with a greater risk of fracture.^20^ Taken together, these results suggest that an increase in BMD reduces the risk of fracture and that combination therapy of denosumab with vitamin D might be optimal.

Antoniucci *et al.* reported that vitamin D status at therapy initiation does not affect the BMD response to ALN when it is co-administered with vitamin D.^21^ Bourke *et al.* reported that baseline dietary intake of calcium and vitamin D status does not alter the effects of zoledronate (ZOL). They concluded that the co-administration of calcium and vitamin D with ZOL may not be necessary for individuals who are not at risk of marked vitamin D deficiency.^22^ Heckman *et al.* reported that, in elderly patients with osteoporosis not responding to BP, vitamin D (1 000 IU daily) may improve BMD at the lumbar spine.^23^ Peris *et al.* reported that an inadequate response to BP treatment is common in postmenopausal women with osteoporosis (as is vitamin D insufficiency) despite vitamin D supplementation.^24^ Roux *et al.* reported that the success of ALN therapy for osteoporosis may be dependent upon vitamin D status.^25^ Whether vitamin D sufficiency or vitamin D administration influences the increased effects of BMD upon BP therapy is controversial.^21–25^ Nevertheless, it is thought that the addition of vitamin D is important if BP treatment is undertaken.

The main limitations of our study were its small sample size and short observation period. Further studies are needed to ascertain whether: (i) BMD continuously increases upon denosumab treatment and to what extent fractures can be prevented; and (ii) the adverse effects (including hypocalcemia) that will occur.

## Conclusions

Hypocalcemia did not occur in the denosumab monotherapy group, but serum calcium decreased only in the denosumab monotherapy group. Thus, vitamin D and calcium should be added when denosumab is administered during osteoporosis treatment. Percent changes in BMD (especially H-BMD) significantly increased in the combination group compared with the denosumab monotherapy group. In addition, we highly recommend vitamin D supplementation with denosumab therapy in patients with primary osteoporosis who are at a high risk of hip fracture.

## Figures and Tables

**Table 1:** Patient characteristics prior to the start of the study

Characteristic	Denosumab monotherapy (*n*=18)	Combination (*n*=23)	*P*-value
Gender (F/M)	15/3	19/4	
Age/years	72.7±2.4	72.7±1.8	0.991 2
BMI/(kg·m^−2^)	22.5±1.0	21.9±0.6	0.646 2
Serum corrected Ca	9.4±0.1	9.1±2.1	0.100 1
Serum phosphorus	3.6±0.1	3.5±0.2	0.494 2
Serum BAP	16.4±2.0	17.2±2.1	0.801 6
Serum TRACP-5b	523.8±60.9	528.9±53.5	0.950 0
Urinary NTX	36.5±6.5	37.6±4.0	0.893 5
1,25(OH)_2_D_3_	53.1±4.5	54.3±4.6	0.853 2
Serum PTH	25.3±2.8	28.3±2.3	0.405 3
BP pre-treatment	8	10	
During of BP use, years	2.1±0.62	2.4±0.57	0.723 9
L1–4 BMD/(g·cm^−2^)	0.793±0.02	0.809±0.03	0.679 5
Total hip BMD/(g·cm^−2^)	0.647±0.02	0.689±0.03	0.168 1

BAP, bone alkaline phosphatase; BMD, bone mineral density; BMI, body mass index; BP, bisphosphonate; NTX, N-terminal telopeptide of type-I collagen; PTH, parathyroid hormone; TRACP-5b, tartrate-resistant acid phosphatase-5b.

Results are the mean±s.e.
